# Ice-Cold Temperature Enhances NADPH Oxidase-Dependent Release of Tissue Factor-Bearing Extracellular Vesicles from Human Monocytic Cells

**DOI:** 10.3390/life16050820

**Published:** 2026-05-15

**Authors:** Akira Nishioka, Toshiharu Azma, Tsutomu Mieda, Yasushi Mio

**Affiliations:** 1 Department of Anesthesiology and Pain Medicine, Kohnodai Hospital, National Center for the Global Health and Medicine, Ichikawa 272-8516, Japan; akiranishiokag@gmail.com; 2 Anesthesiology and Perioperative Medicine, The Jikei University Graduate School of Medicine, Tokyo 105-8461, Japan; 3 Department of Anesthesiology, Saitama Medical University Hospital, Iruma 350-0495, Japan; 4 Department of Anesthesiology, The Jikei University School of Medicine, Tokyo 105-8461, Japan

**Keywords:** tissue factor, phosphatidylserine, monocyte, THP-1, cold storage

## Abstract

The recent rise in whole blood usage for traumatic hemorrhagic shock has renewed interest in the impact of leukocytes on hemostatic function during cold storage. This study investigated whether tissue factor (TF)-bearing extracellular vesicles (EVs) are released from human monocytic cells during cold storage or upon rewarming and whether this process is mechanistically linked to apoptosis. We further examined the contribution of superoxide anion generated by NADPH oxidase (NOX). Methods: THP-1 cells were incubated at 4 °C for up to 24 h with/without test reagents and subsequently rewarmed at 37 °C. Cells were washed by centrifugation before rewarming as required. TF activity in the cell supernatant was quantified, EVs were analyzed by flow cytometry with size-defined gating, and NOX activity normalized to p22*^phox^* was measured by cytochrome c reduction. Results: TF levels and apoptotic cells increased during cold storage. TF release was enhanced 1–2 h after cell lavage following cold exposure, indicating active shedding of TF-bearing EVs rather than passive leakage from damaged membranes. Flow cytometry demonstrated that TF-bearing EVs were distinct from apoptotic vesicles, with a substantial proportion falling within the microvesicle size range. Cold exposure enhanced NOX activity. Both superoxide dismutase (SOD) and catalase inhibited TF release during cold storage; however, only SOD suppressed TF release after cell lavage. Conclusions: TF-bearing EVs are actively shed from human monocytic cells during and after cold storage via a NOX-dependent, superoxide-mediated mechanism. Extracellular SOD suppressed this procoagulant EV release, suggesting a potential strategy to modulate hemostatic alterations associated with cold-stored blood.

## 1. Introduction

Since the first demonstration in 2007 that increasing the ratio of plasma to red blood cell (RBC) transfusion improves survival rates in hemorrhagic trauma care, the association of the balance of plasma/platelets/RBC transfusion with survival rates has been evaluated through clinical studies, including randomized controlled trials [1]. The use of whole blood instead of blood components for massive traumatic hemorrhage has been re-evaluated due to its high hemostatic efficacy.

Furthermore, the enhancement of hemostasis by leukocytes has raised discussions on whether to remove leukocytes, despite the advancements in leukocyte reduction from blood components over the past decades, aimed at preventing transfusion-transmitted viral infections and graft-versus-host disease [2]. On the other hand, in current clinical practice for patients undergoing elective surgery, leukocyte reduction is not performed for autologous blood transfusion products due to the lower risk of infection.

In contrast to the beneficial roles of leukocytes in hemostasis, these cells are also implicated in the formation of thrombi in veins with slow blood flow [3]. In this context, extracellular vesicles released from blood cells have attracted increasing attention as key mediators of procoagulant activity in both physiological and pathological conditions. The molecular mechanisms of leukocyte-mediated hemostatic enhancement include the expression of a negatively charged phospholipid, phosphatidylserine (PS), due to apoptosis, and the production of tissue factor (TF) by monocytes [3,4]. TF-bearing extracellular vesicles from circulating monocytes intensify procoagulant activity in apoptotic cells expressing PS. TF binds coagulation factor VII, triggering the activation of the extrinsic coagulation cascade via the formation of TF/VIIa dimer, which activates factor X in the prothrombinase complex [4]. With the growing use of whole blood in clinical practice, acquiring comprehensive insights into the regulation of procoagulant activities associated with TF is an urgent task. Examining the impact of blood product storage conditions, especially exposure to low temperatures, on these molecular mechanisms is crucial for ensuring safe transfusion medicine.

This study aimed to determine whether TF production in human monocytic cells is induced during cold storage or upon rewarming after storage. Physical stress, including exposure to low temperatures, is known to alter redox homeostasis in leukocytes. Monocytes constitutively express NADPH oxidase (NOX), a major enzymatic source of superoxide anion (·O_2_^−^). Superoxide anion can be further converted into other reactive oxygen species (ROS), such as hydrogen peroxide (H_2_O_2_) and hydroxyl radical (·OH), which are known to participate in apoptosis induction and promote the externalization of PS on the outer leaflet of the cell membrane.

However, the involvement of NOX-derived ROS in TF production by monocytes under cold exposure has not been fully elucidated. In particular, it remains unclear whether TF production is mechanistically linked to apoptosis or occurs through an independent pathway. Accordingly, we assessed the contribution of ROS generated by NADPH oxidase to TF production in human monocytic cells exposed to cold.

## 2. Methods

### 2.1. Materials

RPMI-1640 with phenol red and 2-[4-(2-hydroxyethyl)-1-piperazinyl] ethanesulfonic acid (HEPES) were from Fujifilm Wako Pure Chemical Corporation (Tokyo, Japan). The culture medium used in this study was RPMI-1640, supplemented with 25 mM HEPES, titrated with 2 M NaOH to adjust the pH to 7.4, and stored after passing a 0.2 µm polyether sulfone membrane filter. The culture medium, supplemented with UltraGlutamine (2 mM alanyl L-glutamine, Lonza, Basel, Switzerland), Pen Strep (100 unit/mL penicillin and 100 µg/mL streptomycin, Gibco, Thermo Fisher Scientific, Waltham, MA, USA), and 10% fetal bovine serum (FBS, Biowest, Nuaillé, France), was called as the complete culture medium and was used for passaging human monocytic cells THP-1 (DS Pharma Biomedical (Suita, Osaka, Japan). The culture of THP-1 cells was performed according to the methods previously described [5].

Catalase from bovine liver was purchased from Sigma-Aldrich (St. Louis, MO, USA). Superoxide dismutase (SOD) from bovine erythrocytes and Annexin V-633 or fluorescein isothiocyanate (FITC) apoptosis detection kit were from Nakalai Tesque (Kyoto, Japan). Monoclonal human CD142 antibody conjugated to APC was from Miltenyi Biotec (Auburn, CA, USA). A reduced form of tetrasodium salt β-nicotinamide-adenine dinucleotide phosphate (NADPH) was from Oriental Yeast (Tokyo, Japan). Protease inhibitor cocktail tablets, Roche cOmpete ULTRA were from Merck (Darmstadt, Germany). The CountBright absolute counting beads (approximately 7 µm in diameter) were from Molecular Probes (Eugene, OR, USA). Sodium chloride-sodium phosphate-EDTA (SSPE) tablets (pH 7.4) were purchased from TaKaRa BIO (Ohtsu, Shiga, Japan). The composition of balanced salt solution (BSS, pH 7.4) used in this study was (in mmol/L): NaCl, 127; HEPES, 25; KCl, 4; CaCl_2_, 2; MgCl_2_, 1. The final concentration of Na^+^ in BSS used in this study was adjusted to 140 mM with HEPES-Na or NaCl, respectively. The quality of all chemicals used was certified for molecular biology or for cell culture. Every solution used in this study was membrane-filtered before storage to minimize the background noise in flow cytometry.

### 2.2. General Experimental Protocol (Figure S1)

THP-1 cells were washed three times with the complete culture medium by 200× *g* centrifugation for 2 min. Cell counting was conducted twice using the TC20 automated cell counter (Bio-Rad, Hercules, CA, USA), and the resulting average was used to re-suspend cells in the complete culture medium at a density of 1.5 × 10^5^ cells/mL. These cells in 25 cm^2^ with filter screw caps were subsequently incubated at 37 °C for 24 h in a humidified atmosphere containing 5% CO_2_. After this preconditioning period, flasks were placed for a preselected time in a refrigerator with the temperature controlled at 4 °C in the absence or presence of various reagents. On the other hand, flasks in the control group were incubated at 37 °C in a water bath covered with aluminum foil. After the exposure to cold or the time-matched control incubation, cell suspensions were transferred to each dish of a 96-well multiplate for flow cytometry or to polypropylene microtubes for the measurement of TF. In some experiments, THP-1 cells were washed once with the complete culture medium in the absence or the presence of various reagents before this transfer. A 10-volume percent (vol%) aliquot of fluorescence dye mixtures (Annexin V-633 or -FITC, CD142 antibody and/or propidium iodide (PI)) was mixed with cell suspension (90 vol%) in each dish. The 96-well multiplate containing such mixtures was incubated at 37 °C in a dry bath incubator for a pre-selected time until flow cytometry.

### 2.3. Flow Cytometric Analysis

A 40 µL portion of the cell suspension preloaded with various fluorescence markers and a 4 µL portion of CountBright absolute counting beads (1 × 10^6^ beads/mL) were transferred to a polystyrene tube and filled up to 400 µL with the culture medium, supplemented with 2 mM CaCl_2_. Particles in this tube were analyzed using a FACS Canto II flow cytometer (Becton Dickinson (BD), Franklin Lakes, NJ, USA), according to the methods described previously [5]. The results were primarily processed using BD FACS DIVA software v8.0. Further analyses were performed using Kaluza flow cytometry analysis software ver. 2.1 (Beckman Coulter, Brea, CA, USA).

Forward scatter (FSC), indicative of the relative size of particles, represents light scattered in the same direction as the laser beam and was detected by a photodiode placed at the end of the optical path. Side scatter (SSC), indicative of the relative internal as well as surface complexity of particles, represents light scattered roughly perpendicular to the 488 nm blue laser beam and was detected through a 488/10 nm bandpass filter by a photomultiplier tube. Fluorescence of FITC was measured by excitation with the 488 nm blue laser and detection through a 530/30 nm bandpass filter. Fluorescence of PI was measured by excitation with the 488 nm blue laser and detection through a 585/42 nm bandpass filter. Fluorescence related to APC or Annexin V-633 was measured by excitation with the 633 nm red laser and detection through a 660/20 nm bandpass filter.

The gating strategy used for identifying particles including extracellular vesicles and apoptotic cells was based on forward and side scatter characteristics together with fluorescence signals of Annexin V and TF antibody labeling, as detailed in the Section 3.

### 2.4. Measurement of TF

Cell suspensions from 25 cm^2^ flasks were transferred into individual microtubes and centrifuged at 2400× *g* for 1 min. Supernatants from the microtubes were then transferred to dishes of a 96-well multiplate and stored at −80 °C until the measurement of TF was conducted using an AssaySense human TF chromogenic activity kit (Assaypro LLC, St. Charles, MO, USA), following the manufacturer’s instructions. A 10 µL portion of the supernatant was transferred to another 96-well multiplate, mixed with the assay diluent containing human FVII and FX (70 µL), and incubated at 37 °C for 30 min. A 20 µL portion of the substrate for FXa was then added to each well of the multiplate, and the absorbance at 405 nm was measured every 1 min for 35 min during incubation at 37 °C using a Multiskan Sky plate reader (Thermo Scientific, Waltham, MA, USA).

### 2.5. Preparation of Cell Membrane-Containing Fraction for NOX Activity Measurement

Cell suspension in a 15 mL conical tube was centrifuged at 800× *g* for 2 min. The pellet was resuspended in a 200 µL aliquot of 140 mM sodium/50 mM phosphate buffer (pH 7.4), containing Roche Complete ULTRA protease inhibitors. This cell suspension was gently shaken on ice after the addition of digitonin at a final concentration of 25 µg/mL to permeabilize the cell membrane for 20 min. The resulting pellet was further washed twice with the aforementioned protease inhibitor-containing phosphate buffer by centrifugation at 800× *g* for 2 min. The suspension of this pellet was then disrupted on ice by three cycles of auto-tuned 5 s sonication (10–50 W) with a 2 s pause between each cycle. The resulting cell lysate, containing cell membrane components together with residual nuclei, was transferred to another cryogenic tube. After centrifugation at 800× *g* for 2 min to remove as much nucleic fraction as possible, the supernatant was further transferred to another cryogenic tube. The protein concentration in these cell lysates was measured by optical density at 280 nm. These cryogenic tubes were stored in the vapor phase above liquid nitrogen until the day of the experiments.

### 2.6. Western Blotting

Cell lysates containing the cell membrane fraction were diluted with Bolt LDS sample buffer and reducing agent to adjust the total protein amount to be equal among samples. Proteins were loaded into each lane of sodium dodecyl sulfate (SDS) polyacrylamide gels (Bolt 12% Bis-Tris Plus) and run on the gels at a constant voltage of 200 V for 32 min. The separated proteins were then transferred to membranes using iBlot2 PVDF Mini Stacks (Thermo Fisher Scientific, Waltham, MA, USA).

The membranes were probed with an antibody against p22*^phox^* (1:200 dilution; p22-phox (44.1), Santa Cruz Biotechnology, catalog #sc-130550), followed by a peroxidase-conjugated secondary antibody against mouse IgG (1:5000 dilution; Invitrogen, catalog #G-21040). Protein bands were visualized using Immobilon Forte Western HRP Substrate (Merk, MilliporeSigma, Burlington, MA, USA).

Chemiluminescence emitted from the PVDF membrane was captured using a Nikon Z6 camera equipped with a Nikkor Z 28 mm lens (f/2.8), fixed on a 3D mount in a photo darkroom. The intensity of chemiluminescence from the probed protein was quantified using ImageJ software 1.54g (National Institutes of Health, Bethesda, MD, USA). The relative expression level of p22*^phox^* was determined based on the measured chemiluminescence intensity.

### 2.7. Measurement of NOX Activity

NOX activity was defined as ·O_2_^−^ production from cell lysate containing cell membrane fraction in the presence of NADPH and was measured as the reduction of cytochrome c according to the method described previously with modification [5]. NADPH dissolved in 100 mM Na_2_HPO_4_ (alkaline solution at pH 9.35) was stored in the vapor phase above liquid nitrogen. The concentration of NADPH was measured by optical density at 340 nm (E^mM^_340 nm_ = 6.3) just before the experiment and was further diluted with 100 mM Na_2_HPO_4_ to 1 mM. A 10 µL aliquot of this NADPH-containing Na_2_HPO_4_ was added to a 40 µL aliquot of a mixture containing 50 µg of protein in cell lysate, the oxidized form of cytochrome c, NaH_2_PO_4_ (a counterpart of NADPH-containing Na_2_HPO_4_ to form a 25 mM phosphate buffer at pH 7.4), and SSPE in a disposable plastic cuvette (Eppendorf UVette, Hamburg, Germany) just before the continuous measurement of absorbance at 550 nm using a Multiskan Sky spectrophotometer (Thermo Scientific, Waltham, MA, USA) for 3 min. The final concentrations of components in the 50 µL reaction mixture were as follows: 1 µg/mL protein, 100 µM cytochrome c, 0.2 mM NADPH, 1 mM EDTA, 35 mM PO4^−^(pH 7.4). The reduction rate of cytochrome c was calculated using an extinction coefficient at 550 nm (E^mM^_550 nm_) of 21.

### 2.8. Statistical Analysis

Percentage of apoptotic cells was defined as the number of apoptotic cells divided by the total number of THP-1 cells. The density of vesicles or particles of interest was calculated from the ratio of these particles against CountBright absolute counting beads (1 × 10^6^ beads/mL). These data were shown with a 95% confidential interval (CI). Comparison of ratio or proportion was performed by chi-square test. Data obtained as activities of TF or NOX were expressed as mean ± standard deviation (SD). Comparison of data expressed as mean ± SD among multiple factors with separate levels was performed by analysis of variance (ANOVA) followed by a post hoc Bonferronni’s test. Normality of data distribution was assessed using a Shapiro–Wilk test, and homogeneity of variance among groups was evaluated using Levene’s test before performing ANOVA. Statistical difference was considered significant at a value of *p* less than 0.05.

## 3. Results

### 3.1. TF Released from THP-1 Cells

**Figure 1:**
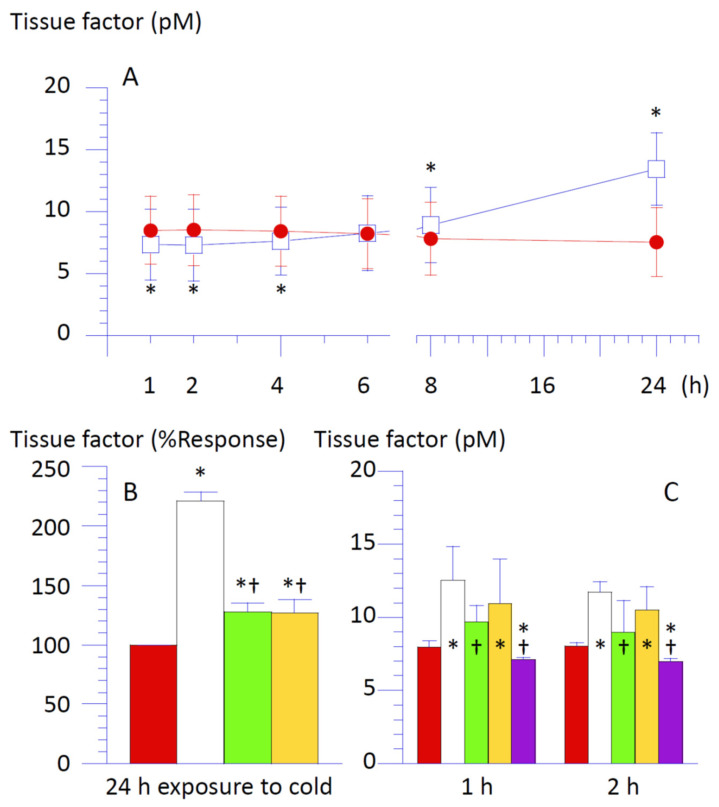
Accumulation of tissue factor in the supernatant of THP-1 cells during cold exposure and following cell lavage. (**A**) Amount of tissue factor (TF, pM) in the supernatant of THP-1 cells incubated for a pre-selected time period at 37 °C (red closed circle) or 4 °C (white open square). (**B**) Levels of TF in the supernatant of THP-1 cells incubated at 4 °C for 24 h in the absence (white) or the presence of SOD (1500 unit/mL, green) or catalase (300 unit/mL, yellow) were expressed as percent of those of cells incubated at 37 °C for 24 h (red). (**C**) Suspension of THP-1 cells incubated at 4 °C for 24 h was rewarmed at 37 °C for 1 h or 2 h after the cells were washed with the complete culture medium by centrifugation at 2400× *g* for 1 min in the absence (white) or the presence of SOD (1500 unit/mL, green), catalase (300 unit/mL, yellow), or EDTA (5 mM, purple). Cell suspension incubated at 37 °C for 24 h (red) underwent centrifugation and washing, similar to the other groups. Levels of TF in the supernatant of THP-1 cells after the rewarming are shown. Data are expressed as mean ± SD (*n* = 8). Post hoc Bonferroni’s test was performed following analysis of variance (*p* < 0.05). * Significantly different from the level of TF in the cell suspension incubated at 37 °C for the same incubation period. † Significantly different from the level of TF in the cell suspension incubated at 4 °C for the same incubation period.

Supernatant from THP-1 cells collected by 2400× *g* centrifugation was collected at specified intervals. These samples were subjected to a chromogenic assay for TF activity (Figure 1). The principle of this assay system is based on the generation of a yellow para-nitroaniline chromophore, which is released from a specific substrate for FXa by its enzymatic activity. This reaction is proportional to the amidolytic activity of the tenase in the extrinsic pathway (TF/FVIIa complex), generating FXa from FX in the test tube. In this assay system, variable amounts of TF in the sample bind to a fixed amount of FVII in the assay kit; thus, the TF/FVIIa complex increases proportionally with the TF concentration in the sample. Due to this property of the assay system, the generation of the end-product chromophore is influenced by contaminated PS on the surface of particles shed from apoptotic cells, as PS attracts coagulation factors X to accelerate their enzymatic activity [4]. Thus, the sample was measured with or without a specific antibody for TF (monoclonal human CD142 antibody, 10% *v*/*v*), and the optical density from the sample in the absence of anti-TF, subtracted by that from the same sample in the presence of anti-TF, was defined as the TF activity in the sample (Supplemental Materials, Table S1).

TF levels in the supernatant of cells exposed to cold for up to 4 h were lower than those of cells incubated at 37 °C throughout for the same time period (i.e., time-matched control). In contrast, exposure equal to or exceeding 8 h elevated TF accumulation, peaking with more than a two-fold increase after 24 h (Figure 1A,B). SOD or catalase suppressed this TF rise (Figure 1B).

### 3.2. Induction of Apoptosis in THP-1 Cells Exposed to Cold

**Figure 2:**
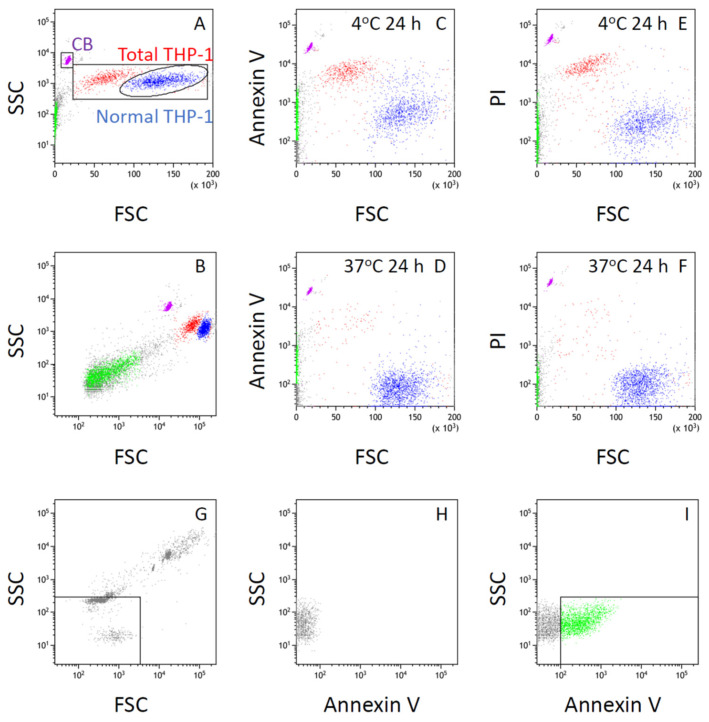
Flow cytometric characterization and gating of particles in THP-1 cell suspension. Suspension of THP-1 cells was incubated at 4 °C for 24 h, followed by rewarming at 37 °C for 1 h, and then subjected to flow cytometry after staining with Annexin V–FITC and propidium iodide (PI). (**A**) Gates for normal and total THP-1 cells in the plot of side scatter (SSC) versus forward scatter (FSC) are shown and indicated in blue and red, respectively. The gate for CountBright beads (CB) is also shown and indicated in violet. (**B**) Same as (**A**), shown with a logarithmic FSC scale. (**C**) FITC fluorescence (Annexin V–FITC) versus FSC of particles in the same cell suspension as in (**A**,**B**). (**D**) Same as (**C**), except that the suspension was incubated at 37 °C as a time-matched control for cold exposure. (**E**) PI fluorescence versus FSC of particles in the same cell suspension as in (**C**). (**F**) Same as (**E**), except that the suspension was incubated at 37 °C as a time-matched control. (**G**) FSC–SSC plot obtained from size-calibration beads for microvesicles (Megamix) mixed with 1 vol% CountBright beads, used to define the gate for microvesicle-sized particles. (**H**) THP-1 cell suspension derived from the same sample as in (**A**–**C**,**E**) (incubated at 4 °C), without Annexin V staining. Only particles within the gate for microvesicle-sized particles are shown. (**I**) FITC fluorescence (Annexin V–FITC) of particles in the same cell suspension as in (**A**–**C**,**E**), with analysis restricted to particles within the gate for microvesicle-sized particles. Annexin V–positive particles (square gate) are indicated in green. The corresponding particles in (**A**–**C**,**E**) are also highlighted in green.

**Figure 3:**
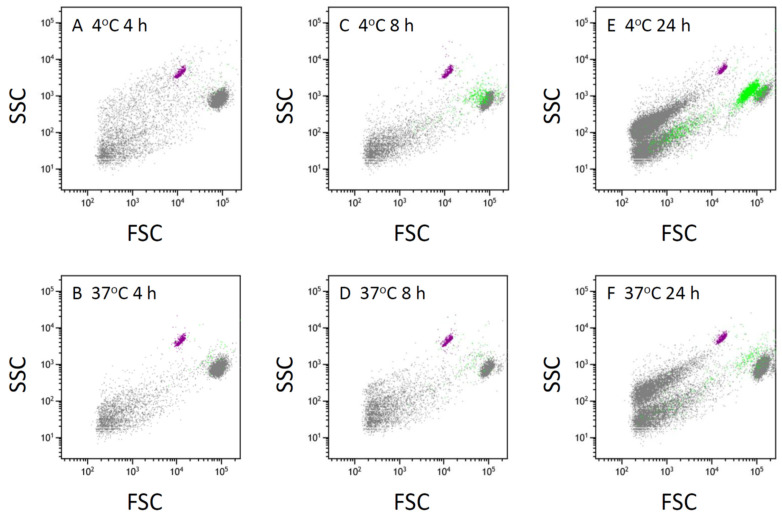
Time-dependent increase in phosphatidylserine-positive cells and vesicle-sized particles in THP-1 cell suspension. Suspension of THP-1 cells incubated at 4 °C or 37 °C for 4 h (**A**,**B**), 8 h (**C**,**D**), or 24 h (**E**,**F**), followed by rewarming at 37 °C for 1 h, was subjected to flow cytometry after staining with Annexin V–633. Gating strategy, including the identification of THP-1 cells and CountBright beads, was defined as described in Figure 2. Cells and vesicle-sized particles positive for Annexin V–633 are indicated in green, reflecting phosphatidylserine exposure.

The suspension of THP-1 cells exposed to cold was collected at the same time intervals as the supernatant and was mixed with Annexin V, a marker for PS that is known to be expressed on the surface of apoptotic cells or apoptosis-related particles. This entire cell suspension was then subjected to flow cytometry (Figure 2 and Figure 3).

The suspension of THP-1 cells consisted of three groups of particles in the FSC–SSC plot (Figure 2A,B). The majority of particles in the FSC–SSC plot were normal THP-1 cells, which showed the largest FSC among all particle groups (Figure 2A). Normal THP-1 cells were accompanied by a second large group of cells whose FSC range was smaller than that of normal THP-1 cells, whereas their SSC was higher than that of normal THP-1 cells at comparable FSC values (Figure 2A,B). It is generally known that FSC indicates the size of cells or particles in flow cytometry, whereas SSC reflects the surface as well as the internal complexity of each particle. Therefore, the second large group of particles was considered to possess properties consistent with apoptotic cells, which are smaller than normal cells of their origin and contain fragmented nuclei.

Our previous studies indicated that this second large group of particles was stained with the fluorescent nuclear dye Hoechst 33342 [5], whereas staining with tetramethylrhodamine methyl ester (TMRM) was weaker than that of normal THP-1 cells [6]. Because TMRM fluorescence reflects mitochondrial membrane potential as well as the abundance of intact mitochondria within cells or vesicles [7], these findings suggest that this second large group of particles possesses characteristics of apoptotic cells. Our previous studies also indicated that only particles with FSC values larger than those of the CountBright counting beads were stained with Hoechst 33342 [5]. In this study, therefore, the gate for “total THP-1 cells” was defined as a square with FSC values between the maximum FSC of the CountBright beads and the maximum FSC of normal THP-1 cells, and SSC values between the minimum and maximum SSC of normal THP-1 cells or the second large group of particles (Figure 2A).

The proportion of the second large group of cells among total THP-1 cells exposed to cold increased in a time-dependent manner (Figure 3). These cells were strongly stained with Annexin V, a marker for PS expressed on the surface of apoptotic cells or vesicles (Figure 2C,D and Figure 3). The same cells were also stained with propidium iodide (PI), which intercalates into double-stranded DNA and therefore indicates increased membrane permeability to this cationic dye (Figure 2E,F). These findings suggest that the second large group of cells, which were smaller than normal THP-1 cells but exhibited higher SSC values, correspond to late apoptotic cells.

The mean fluorescence level related to Annexin V in normal THP-1 cells exposed to cold for 24 h was higher than that in normal THP-1 cells without cold exposure (Figure 2C,D). A portion of these Annexin V-positive cells was also stained with PI (Figure 2C,E), suggesting that exposure of THP-1 cells to cold for up to 24 h induced apoptotic changes ranging from early to late stages in the population of normal THP-1 cells.

The FSC values of these THP-1 cells were larger than those of the CountBright fluorescence calibration beads (CB in Figure 2A, approximately 7 µm). In contrast, particles with FSC values smaller than those of THP-1 cells were also detected in the FSC–SSC plot (Figure 2B). These small particles increased in a time-dependent manner during incubation (Figure 3). Because their FSC values were smaller than those of the CountBright beads (Figure 2B) and they were not potently stained with the nuclear dye Hoechst 33342 in our previous studies [5], these particles were considered to represent cell-derived vesicles or subcellular fragments rather than intact THP-1 cells. Exposure of the THP-1 cell suspension to cold further enhanced this time-dependent increase in smaller particles (Figure 3).

The proportion of Annexin V-positive THP-1 cells, indicating cell surface expression of PS, in total THP-1 cells exposed to cold for 4 h did not significantly differ from that in cells incubated at 37 °C for the same time period (time-matched control) (Figure 3A,B, Table 1). However, after 8 h of cold exposure, the proportion of PS-positive cells was significantly higher than in the time-matched control (Figure 2C,D, Table 1). This proportion further increased in the cell suspension exposed to cold for 24 h, compared to those exposed for 8 h (Figure 2C–F, Table 1).

### 3.3. TF Release from THP-1 Cells After Lavage Following Cold Exposure

Timing of the inversion of TF levels in the supernatant of THP-1 cells with or without exposure to cold (Figure 1A) followed the emergence of PS-positive cells after cold exposure (Figure 3, Table 1). To assess whether increased TF resulted from TF leakage from cell membrane during apoptosis, cells exposed to cold for 24 h were washed with complete culture medium by centrifugation at 2400× *g* for 1 min. Preliminary experiments demonstrated that THP-1 cells were separated from particles shed from these cells by the centrifugation at this gravity for at least 30 s, thus indicating that TF released during the exposure to cold was removed from the cell suspension by this cell lavage.

TF levels in the replaced medium collected after rewarming at 37 °C for 1 h or 2 h following 24 h cold exposure significantly increased compared to the control (Figure 1C). Accumulated TF at 2 h post-lavage was not higher than that at 1 h (Figure 1C), suggesting rapid TF release (<1 h) in response to certain stimuli, such as cell lavage using pipetting and centrifugation. Figure 1A also supports this concept because TF from THP-1 cells incubated at 37 °C throughout (control) reached its maximum level at 1 h after the cell preparation, remaining stable during the 24 h observation. This suggested that THP-1 cells do not secrete TF constantly.

The addition of SOD (1500 U/mL) to the cell suspension significantly reduced TF accumulated after cell lavage and rewarming following 24 h cold exposure compared to cells without SOD (Figure 1C). Calcium chelators, EDTA (Figure 1C) or citrate, added instead of SOD, further suppressed TF accumulation in the same setting. TF in the EDTA-containing medium was lower than that from THP-1 cells without exposure to cold (Figure 1C). Catalase, replacing SOD, did not significantly impact TF accumulation (Figure 1C). Taken together, calcium influx through the cell membrane is likely responsible for prompt and transient TF release from THP-1 cells provoked by certain reproduced stimuli, such as experimental manipulations. Exposure to cold enhances TF release, confirming that ·O_2_^−^ is a direct contributing factor because SOD, but not catalase, suppresses this enhancement even without calcium chelators.

### 3.4. Localization of TF and PS on Particles Shed from THP-1 Cells

**Figure 4:**
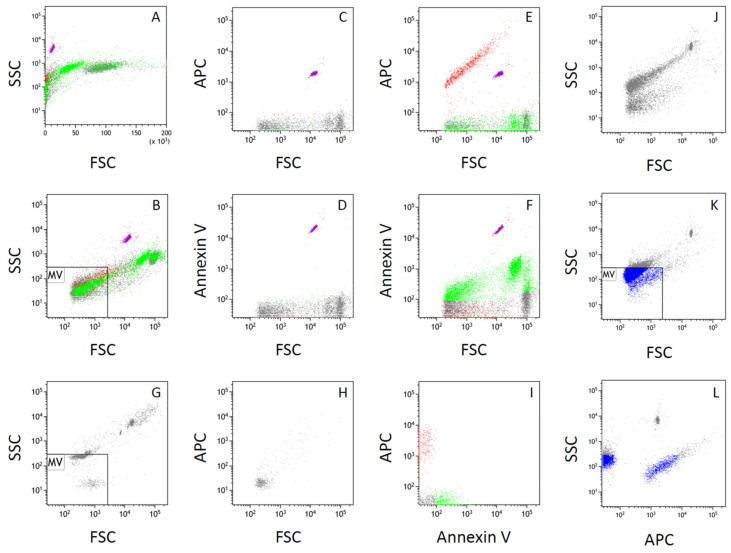
Distinct localization of tissue factor and phosphatidylserine on vesicles, with identification of TF-bearing vesicles within the microvesicle size range. (**A**) Suspension of THP-1 cells shown in the plot of side scatter (SSC) versus forward scatter (FSC). Cells were incubated at 4 °C for 24 h, followed by rewarming at 37 °C for 1 h, and then subjected to flow cytometry after staining with anti-CD142 (tissue factor) antibody conjugated with APC and Annexin V–FITC. (**B**) Same as (**A**), shown with a logarithmic FSC scale. Gating strategy, including the identification of CountBright beads and microvesicle-sized particles (MV), was defined as described in Figure 2. (**C**) APC fluorescence versus FSC from particles in the THP-1 cell suspension derived from the same sample as in (**A**,**B**,**E**,**F**), without staining with anti-CD142 antibody or Annexin V, showing background APC fluorescence. (**D**) FITC fluorescence versus FSC from the same cell suspension as in (**C**), showing background FITC fluorescence. (**E**) APC fluorescence versus FSC from the same cell suspension as in (**A**,**B**). (**F**) FITC fluorescence versus FSC from the same cell suspension as in (**A**,**B**). (**G**) FSC–SSC plot obtained from size-calibration beads for microvesicles (Megamix) mixed with 1 vol% CountBright beads, used to define the gate for microvesicle-sized particles (MV). (**H**) FSC–SSC plot obtained from culture medium containing 2 mM CaCl_2_. (**I**) APC fluorescence versus FITC fluorescence from the same cell suspension as in (**A**,**B**,**E**,**F**), with analysis restricted to particles gated as MV. (**J**) FSC–SSC plot of particles in the suspension derived from the same sample as in (**A**,**B**,**E**,**F**) after centrifugation at 2400× *g* for 1 min. (**K**) The suspension in (**J**) was further centrifuged at 12,000× *g* for 15 min to sediment vesicles. FSC–SSC plot of particles in the pellet resuspended in filtered culture medium containing 2 mM Ca^2+^ and incubated with anti-CD142 antibody conjugated with APC (10 vol%). (**L**) SSC versus APC fluorescence of particles from the same suspension as in (**K**). Particles gated as MV are indicated in blue.

Incubation of the THP-1 cell suspension with a specific antibody against TF (CD142) and Annexin V, a specific marker for PS, emerging on the surface of apoptotic cells or particles, revealed that neither normal nor apoptotic THP-1 cells expressed TF on the cell surface. However, a portion of particles present in the cell suspension was stained with anti-CD142 conjugated with APC (Figure 4A–F). A plot of APC-related fluorescence versus FSC showed that CD142-positive particles consisted of those within a range from the detection threshold of FSC to those larger than the CountBright beads (Figure 4C,E). A plot of Annexin V-FITC-related fluorescence versus FSC also showed that PS-positive particles consisted of those among a wide range including the detection threshold of FSC (Figure 4D,F).

Although FSC is related to the size of particles in flow cytometry, particles with FSC values close to the detection limit do not necessarily reflect the true particle size. For such small particles, the height of the electric pulse for SSC has been suggested to correlate more closely with particle size than the area of the electric pulse for FSC.

The solution of size calibration beads for microvesicles (Megamix), mixed with 1 vol% CountBright beads, indicated that the FSC value of the 3 µm beads appeared smaller than that of the 7 µm CountBright beads, whereas the 0.5, 0.9, and 3 µm beads in the Megamix could not be clearly separated on the FSC scale (Figure 2G and Figure 4G). In contrast, these beads were more clearly separated according to size on the SSC scale (Figure 2G and Figure 4G).

From the FSC–SSC plot obtained from the Megamix solution, the gate for microvesicle-sized particles was defined as a square, with the maximum FSC corresponding to that of the 0.5 µm beads and the maximum SSC corresponding to that of the 0.9 µm beads (Figure 2G and Figure 4G).

Figure 2B,G–I demonstrated that a portion of Annexin V-positive particles was included in this gate, suggesting that part of the PS-positive particles were within the microvesicle size range. Table 2 shows that PS-positive microparticles increased in a time-dependent manner following cold exposure, consistent with the increase in PS-positive THP-1 cells (Table 1).

The SSC value of the 0.5 µm beads in the Megamix was comparable to that obtained from culture medium containing 2 mM CaCl_2_ that had been passed through a 0.2 µm filter after addition of CaCl_2_ (Figure 4G,H). These findings suggest that particles smaller than approximately 0.5 µm could not be reliably distinguished from background signals under the present experimental conditions.

Figure 4I shows that a portion of CD142-positive particles, as well as PS-positive particles, was included within this gate. Taken together with the finding that CD142-positive particles appeared across a broad range of FSC values, including those larger than the CountBright beads (Figure 4E), these results suggest that TF-bearing particles include both larger particles comparable to apoptotic cells or vesicles and smaller particles classified as microvesicles.

To confirm that a portion of TF-bearing particles are present as microvesicles, the suspension of THP-1 cells exposed to cold for 24 h was centrifuged at 2400× *g* for 1 min to obtain the supernatant used for the TF activity assay (Figure 4J). This centrifugation step was intended to remove intact cells and large cellular debris while retaining smaller vesicles in the supernatant. The FSC–SSC plot of this supernatant indicated that THP-1 cells and apoptotic cells, but not CountBright beads, were removed by this step (Figure 4J).

The supernatant was further centrifuged at 12,000× *g* for 15 min to sediment vesicles. The resulting pellet was resuspended in filtered culture medium containing 2 mM Ca^2+^ and incubated with anti-CD142 antibody conjugated with APC (10 vol%) (Figure 4K,L). After 10 min at room temperature, the suspension was subjected to flow cytometry. The APC–SSC plot demonstrated that the supernatant obtained after 2400× *g* centrifugation contained CD142-positive vesicles (Figure 4L). Based on the proportion of vesicles within the microvesicle gate, 77.1% of CD142-positive vesicles were classified as microvesicles (95% confidence interval, 74.5–79.4%; *n* = 1107; Figure 4L).

Because small extracellular vesicles are expected to remain in the supernatant after centrifugation at 12,000× *g*, the TF-bearing vesicles detected in the present study are unlikely to consist predominantly of exosome-sized vesicles or soluble proteins secreted via exocytosis [8].

Together with the chromogenic assay results, in which the generation of the para-nitroaniline chromophore significantly differed between paired supernatant samples with or without CD142 antibody (Table S1), these findings suggest that procoagulant TF activity resides, at least in part, in TF-bearing microvesicles. These vesicles are distinct from PS-positive particles related to apoptosis (Figure 4I), indicating that the mechanisms responsible for shedding TF-bearing vesicles are separate from those generating apoptosis-related particles.

### 3.5. Western Blotting

A key finding in this study was that SOD suppressed TF release enhanced by exposing THP-1 cells to cold. SOD catalyzes the dismutation of superoxide anion (·O_2_^−^) to hydrogen peroxide (H_2_O_2_) [9], suggesting that ·O_2_^−^ rather than H_2_O_2_ is the primary reactive oxygen species involved in TF release following cold exposure. Because SOD used in this study is a homodimeric protein of approximately 16.3 kDa [10,11], it is likely that SOD added to the cell suspension localized predominantly at the outer leaflet of the cell membrane.

Monocytes express the NADPH oxidase (NOX) family member gp91*^phox^* (NOX2), which is embedded in the lipid bilayer of the cell membrane and forms a heterodimer with p22*^phox^*, another membrane-associated subunit essential for NOX stability and function [12]. The gp91*^phox^*–p22*^phox^* complex transfers electrons from NADPH on the cytoplasmic side of the membrane to molecular oxygen on the extracellular side, resulting in the generation of ·O_2_^−^ at the cell surface.

**Figure 5:**
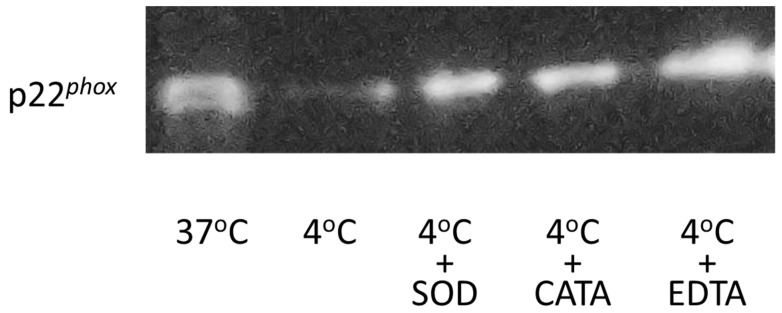
Expression of p22*^phox^* in the cell membrane fraction. Proteins from cell lysates containing the cell membrane fraction were separated by SDS–PAGE (Bolt 12% Bis-Tris Plus) at a constant voltage of 200 V for 32 min under reducing conditions. Equal amounts of total protein were loaded in each lane. Uncropped images of the blots are provided as Supplementary Figures (Figures S2A,B).

Western blot analysis of the cell membrane fraction demonstrated that the expression level of p22*^phox^* was significantly reduced in THP-1 cells exposed to cold (4 °C) for 24 h, reaching approximately 40–60% of that observed in cells maintained without cold exposure (Figure 5, Table 3). The addition of SOD (1500 units/mL), catalase (300 units/mL), or EDTA (5 mM) effectively inhibited the cold-induced reduction of p22*^phox^* in the cell membrane fraction.

Given that p22*^phox^* serves as an essential structural component of the NOX complex at the cell membrane, these findings indicate that cold exposure alters the membrane-associated NOX machinery in THP-1 cells. Notably, the inhibitory effects of catalase and EDTA on p22*^phox^* reduction contrast with their limited effects on TF release observed in earlier experiments. This discrepancy suggests that changes in p22*^phox^* expression during cold exposure may reflect structural alterations of the cell membrane associated with apoptosis, which is known to be enhanced under cold storage conditions.

### 3.6. NOX Activity in Cell Lysate

NOX activity in the cell membrane fraction was assessed by measuring the reduction of cytochrome c in the presence of NADPH. When the amount of membrane fraction was normalized by total protein concentration, NOX activity in lysates from THP-1 cells incubated at 4 °C for 24 h appeared lower than that observed in lysates from cells maintained without cold exposure.

However, as shown by Western blot analysis, the amount of p22*^phox^* in the cell membrane fraction was markedly reduced by cold exposure (Table 3). Because p22*^phox^* is an essential structural component of the functional NOX complex, normalization of NOX activity solely by total protein content likely underestimated NOX activity in viable cells within cold-exposed samples, which contained a substantial proportion of apoptotic cells (Table 1 and Figure 2 and Figure 3). Apoptosis-associated membrane disruption may reduce detectable membrane-bound p22*^phox^* independently of intrinsic NOX enzymatic activity.

Therefore, NOX activity in the membrane fraction was adjusted according to the level of p22*^phox^* in each sample, calculated based on the relative chemiluminescence intensity obtained from Western blotting using a p22*^phox^*-specific antibody (Table 3). After normalization to p22*^phox^* expression, NOX activity in THP-1 cells exposed to cold for 24 h was significantly higher than that in cells without cold exposure (Table 4), indicating that superoxide-generating activity per unit of membrane-associated NOX was enhanced in the remaining viable cell population despite the overall reduction in p22*^phox^* expression caused by apoptosis.

The addition of SOD (1500 units/mL) to the cell lysate significantly inhibited NOX activity, whereas increasing the SOD concentration to 3000 units/mL did not result in further suppression compared with 1500 units/mL. This indicates that 1500 units/mL of SOD was sufficient to scavenge ·O_2_^−^ in the experimental system (Table 4). In contrast, catalase (300 units/mL) did not significantly affect NOX activity compared with the absence of catalase, suggesting that NOX-derived ·O_2_^−^ production itself was not directly influenced by hydrogen peroxide, while the apparent effects of catalase observed in membrane-associated parameters (i.e., expression of p22*^phox^* in membrane fraction) may be attributable, at least in part, to apoptosis-related membrane alterations rather than direct modulation of NOX enzymatic function.

## 4. Discussion

### 4.1. Localization of TF Produced by Human Monocytic Cells

The TF localization in human monocytic cells has not yet been extensively studied. Conde et al. reported TF enrichment of microvesicles shed from monocytes stimulated with lipopolysaccharide (LPS) and THP-1 cells with or without stimulation, with confirmed localization in lipid rafts using density gradient ultracentrifugation of detergent-treated cell fractions [13]. They speculated that TF-bearing vesicles bud similarly to enveloped viruses, like human immunodeficiency virus (HIV), which fall within the size range of microvesicles. In their study, “microvesicles” were collected from the supernatant after centrifuging the cell suspension at 5000× *g*. However, their technique suggests inclusion of vesicles larger than microvesicles (>1 µm) in their samples.

Lewis et al. observed, using immunogold electron microscopy, that pigeon monocytes stimulated by LPS or oxidized low-density lipoprotein elaborated ruffles and microvilli on the cell membrane, where TF antigen was localized [14]. Nishimura et al. recently explored mechanisms related to the generation of large extracellular vesicles [15]. It is known that membrane curvature is formed by bin-amphiphysin-Rvs (BAR) family proteins, among which inverse BAR proteins are involved in the protrusion of filopodia. They demonstrated that inverse BAR proteins including missing in metastasis (MIM) induce vesicle formation by scission of filopodia, a process enhanced by external forces. This aligns with our suggestion that TF-bearing vesicle shedding may be induced by experimental manipulations consistently employed in our study.

In this study, we confirmed that TF-bearing particles shed from human monocytic THP-1 cells were similar in size to apoptotic particles, based on flow cytometric analysis with Annexin V as a marker for PS, although TF-bearing vesicles themselves did not bind Annexin V. This finding suggests that TF-bearing vesicles are generated as large extracellular vesicles, similar to apoptotic vesicles. Additionally, using flow cytometry with fluorescence-labeled antibodies, we were able to distinguish these TF-bearing vesicles, identified by specific TF antibodies, from particles related to apoptosis. This distinction was further supported by flow cytometric analyses using FSC–SSC gating defined with size calibration beads, which demonstrated that a substantial proportion of TF-bearing vesicles fell within the microvesicle size range, while remaining distinct from Annexin V-positive particles.

Because TF and PS are both recognized as key contributors to the procoagulant activity of extracellular vesicles, they are often suggested to coexist on the same vesicles. However, to our knowledge, there is no clear evidence in the literature supporting the coexistence of TF and PS on the same extracellular vesicles. In the current study, TF presence was evaluated both by its enzymatic activity in a chromogenic assay and by fluorescence-labeled flow cytometry. The significant reduction in TF activity upon the addition of a specific TF antibody indicates that the molecule bound to the TF antibody possesses tenase activity and that this enzymatic activity is localized on extracellular vesicles distinct from those containing PS. Therefore, we believe this study provides the first evidence that TF-bearing vesicles shed from human monocytic cells are distinct from apoptotic particles.

### 4.2. Role of Superoxide-Centered Redox Signaling in Cold-Induced TF Release

In the present study, SOD inhibited TF release from THP-1 cells during both the 24 h incubation at 4 °C and the subsequent cell lavage.

ROS, including ·O_2_^−^, H_2_O_2_, and ·OH, are known to play pivotal roles in redox signaling [9]. For example, ·OH generated through the Haber–Weiss cycle from H_2_O_2_ activates calcium influx in endothelial cells, while the addition of a reducing agent, dithiothreitol, alleviates calcium influx through the cell membrane calcium channels [16]. In situations where ·OH is the responsible ROS for redox signaling, SOD is ineffective as it forms H_2_O_2_ from ·O_2_^−^ [9,16]. On the other hand, catalase inhibits redox signaling by decreasing H_2_O_2_, the substrate of ·OH in the Haber–Weiss cycle [16]. Therefore, we suggest that ·O_2_^−^, rather than H_2_O_2_ or ·OH, is the primary ROS contributing to the enhanced TF release induced by the exposure of THP-1 cells to cold.

While ·O_2_^−^ is considered less reactive than ·OH or its substrate H_2_O_2_, experimental evidence indicates that ·O_2_^−^ serves as a redox signal for certain proteins like aconitase, containing a [4Fe-4S] cluster [17]. Ferrous iron is removed from this [4Fe-4S] cluster by ·O_2_^−^, leading to aconitase inactivation [18]. Gardner and Fridovich demonstrated that SOD addition increased aconitase activity in samples from SOD-deficient Escherichia coli, highlighting ·O_2_^−^ as a primary ROS for redox signaling [17]. The precise molecular mechanism to modulate TF release from THP-1 cells remains unclear in this study. However, our findings provide initial evidence for the involvement of ·O_2_^−^ derived from NOX and extracellularly localized SOD in TF release from human monocytic cells. Notably, after normalization to membrane-associated p22*^phox^* levels, NOX activity per unit enzyme was increased in cold-exposed cells (Table 4), supporting the involvement of enhanced superoxide generation in this process.

In contrast to the inhibitory effects of SOD on TF release from THP-1 cells during both the 24 h incubation at 4 °C and the subsequent cell lavage, catalase inhibited TF release only during the 24 h incubation. H_2_O_2_, known as a long-lasting oxidant, reaches higher concentrations compared to other short-lived oxidants like ·O_2_^−^ or ·OH [9]. Therefore, we suggest that the inhibitory effects of catalase on the enhanced TF release from THP-1 cells after cold exposure depend on the duration of ·O_2_^−^ generation from NOX. This is because H_2_O_2_, converted from ·O_2_^−^ [9] and accumulated in the supernatant of THP-1 cells after cell lavage (1–2 h), is expected to be smaller than that generated during the 24 h exposure to cold.

Although catalase did not suppress TF release after cell lavage and rewarming, its inhibitory effect during the 24 h cold exposure suggests an additional mechanism distinct from direct regulation of TF-bearing vesicle shedding by ·O_2_^−^. While this study did not directly assess apoptotic signaling pathways, the observed effects of catalase, together with those of calcium chelation by EDTA, raise the possibility that hydrogen peroxide-dependent apoptosis was partially attenuated during prolonged cold exposure.

Cell apoptosis is commonly triggered through either extrinsic or intrinsic pathways [9]. Extrinsic apoptotic pathways initiate via the activation of cell surface receptors, such as Fas ligand and TNF-α, while intrinsic apoptosis is provoked, independent of such receptors, by various stimuli, including toxins, viral infections, free radicals, and thermal conditions [9]. Intrinsic apoptosis is also known to be closely associated with mitochondrial dysfunction [9,19]. ROS are known to promote mitochondrial outer membrane permeabilization, cytochrome c release, and subsequent activation of downstream apoptotic cascades [9]. In addition, calcium overload in mitochondria facilitates the opening of the mitochondrial permeability transition pore, further enhancing cytochrome c release [7]. Previous studies have highlighted H_2_O_2_ as a key mediator of apoptosis due to its relative stability and ability to function as a signaling molecule regulating mitochondrial pathways and caspase activation [9].

Therefore, we suggest that the suppressive effects of catalase and EDTA observed during cold exposure may reflect partial inhibition of ROS- and calcium-dependent apoptotic processes, which indirectly influence membrane integrity and NOX-associated parameters, rather than direct modulation of TF release mechanisms.

### 4.3. Implication for Whole Blood Transfusion

Allogenic whole blood transfusion is currently emerging as an alternative to blood component therapy for traumatic hemorrhagic shock [1,20]. This marks a revolutionary shift in the landscape of damage control resuscitation, driven by the understanding of disseminated intravascular coagulation (DIC) in trauma patients [1].

This paradigm departs from the traditional concept of dilutional coagulopathy, which typically becomes evident only after blood loss exceeds one blood volume (i.e., total volume of circulation blood in a human body), and has been largely informed by clinical experience in elective surgery [21]. The practical use of stored whole blood containing white blood cells is currently a prominent clinical question, particularly concerning whether hemostasis is improved [2,20], especially during trauma care. From the perspective of trauma resuscitation, the presence of leukocytes in stored whole blood may contribute positively to hemostasis, partly through procoagulant mechanisms such as TF-dependent pathways.

In contrast to the packages used for the blood component therapy where leukocytes are removed by filtration, white blood cells are not depleted from the package of autologous whole blood transfusion stored before elective surgery. Concerning the risk of venous thrombosis in elective surgery, which is generally associated with a lower risk of massive bleeding, monocyte-derived TF may have unfavorable effects [3]. In particular, TF-bearing vesicles generated during storage of autologous whole blood may increase the thrombotic risk in these patients. Therefore, understanding the mechanisms that induce TF release in human monocytes during and after cold storage is a current clinical interest that needs elucidation.

The findings of this study suggest that calcium chelation is effective in preventing TF release from monocytic cells both during cold storage and after rewarming from the storage condition (Figure 2). However, in clinical practice, calcium chelation cannot be maintained during transfusion, limiting its effectiveness in preventing monocytic cells from forming TF-bearing vesicles.

Taken together, preservation strategies that maintain leukocyte-containing whole blood under calcium-chelated conditions may be beneficial in trauma settings, whereas the same mechanisms may increase thrombotic risk in patients undergoing elective surgery who primarily require correction of anemia rather than enhancement of coagulation.

In contrast, the extracellular addition of SOD effectively suppressed TF release from THP-1 cells during both cold storage and rewarming. The implications of our findings extend to the search for ideal additives for storing whole blood containing monocytes.

### 4.4. Limitations Related to the Use of THP-1 Cells

In this study, we used THP-1 cells as a model of human monocytic cells to investigate the mechanisms underlying TF release and vesicle formation. THP-1 is an acute monocytic leukemia-derived cell line [22] and therefore does not fully represent the physiological properties of primary human monocytes. In particular, the responses of THP-1 cells to environmental stimuli, including cold exposure and redox modulation, may differ quantitatively or qualitatively from those of native monocytes.

However, the present study was designed as a hypothesis-generating investigation to address fundamental and unresolved questions, including whether TF and PS are localized on the same or distinct vesicle populations, and whether superoxide-dependent mechanisms are involved in TF release. Given the lack of prior evidence directly examining these issues [3], a controlled and reproducible experimental system was required to dissect these mechanisms.

Our findings demonstrate that TF-bearing vesicles are distinct from PS-positive particles, thereby challenging the commonly accepted assumption that these procoagulant components coexist on the same extracellular vesicles [3]. These observations provide a conceptual basis for future studies using primary human monocytes or clinical samples.

Further investigations using human monocytes derived from peripheral blood will be necessary to validate the physiological relevance of these findings and to determine their clinical implications in the context of blood storage and transfusion.

## 5. Conclusions

In conclusion, we demonstrated that TF-bearing vesicles shed from human monocytic cells are distinct from apoptotic particles, that TF release is regulated by NOX-derived ·O_2_^−^, and that extracellularly localized SOD effectively suppresses this procoagulant vesicle shedding during and after cold storage.

## Figures and Tables

**Table 1:** Changes in the proportion of phosphatidylserine-positive THP-1 cells by the exposure to cold.

		4 °C				37 °C	
		PS-Positive Cells				PS-Positive Cells	
	(*n*)	(%)	**95% CI**		(*n*)	(%)	**95% CI**
4 h	(3607)	0.7%	(0.5–1.1)	NS	(3732)	0.5%	(0.3–0.8)
8 h	(3906)	7.9%	(7.1–8.8)	*	(2382)	2.5%	(1.9–3.2)
24 h	(3575)	53.8%	(52.2–55.5)	*	(6992)	1.9%	(1.6–2.2)

THP-1 cells were incubated at 4 °C or 37 °C for 4 h, 8 h, or 24 h. *n* = number of cells counted in flow cytometry. * Significantly different from the proportion of phosphatidylserine (PS)-positive THP-1 cells (%) incubated at 37 °C for the same time period (*p* < 0.01, chi-square test). NS, not significant. CI, confidential interval.

**Table 2:** Changes in the number of phosphatidylserine-positive microparticles shed from THP-1 cells by the exposure to cold.

	4 °C			37 °C	
	PS-Positive Microvesicle Sized Particles			PS-Positive Microvesicle Sized Particles	
	×10^3^ counts/mL	95% CI		×10^3^ counts/mL	95% CI
4 h	1.6	(0.9–2.7)	NS	1.5	(0.8–2.6)
8 h	6.6	(5.1–8.6)	*	3.9	(2.7–5.7)
24 h	51.0	(47.0–55.0)	*	9.0	(7.2–11.1)

THP-1 cells were incubated at 4 °C or 37 °C for 4 h, 8 h, or 24 h. * Significantly different from the number of phosphatidylserine (PS)-positive microparticles (×10^3^ counts/mL) in the suspension of THP-1 cells incubated at 37 °C for the same time period (*p* < 0.05, chi-square test). NS, not significant. CI, confidential interval.

**Table 3:** Chemiluminescence from separated proteins in the cell membrane fraction.

**Expression of p22*^phox^***		(*n*)		
	37 °C	(4)	100%	
	4 °C	(4)	56 ± 10%	*
	+ SOD, 1500 unit/mL	(4)	112 ± 20%	NS
	+ catalase, 300 unit/mL	(4)	102 ± 16%	NS
	+ EDTA, 5 mM	(4)	132 ± 26%	NS

The intensity of chemiluminescence from each band, probed by specific antibodies, was captured and quantified by densitometry. “*n*” represents the number of Western blots analyzed. * Significantly different from the blot in the lane loaded with proteins obtained from THP-1 cells without exposure to cold for 24 h (i.e., vs. 37 °C). NS, not significantly different from 37 °C (*p* < 0.05).

**Table 4:** NADPH oxidase (NOX) activity in the cell membrane fraction.

Reduction of Cytochrome c					
		(nM/µg/min)	(*n*)	Mean ± SD	
	37 °C		(6)	47.7 ± 6.7	
		+ SOD, 1500 unit/mL	(6)	30.4 ± 3.7	*p* < 0.01 (vs. 37 °C)
		+ SOD, 3000 unit/mL	(6)	29.3 ± 1.3	*p* < 0.01 (vs. 37 °C), *NS* (vs. 1500 unit/mL SOD)
		+ catalase, 300 unit/mL	(6)	47.1 ± 3.8	*NS* (vs. 37 °C)
	4 °C		(6)	70.8 ± 8.8	*p* < 0.01 (vs. 37 °C)

Data are expressed as mean ± SD. “*n*” represents the number of experiments performed. NS, not significant.

## Data Availability

The original contributions presented in this study are included in the article/Supplementary Material. Further inquiries can be directed to the corresponding author.

## Supplementary Materials

The following supporting information can be downloaded at: https://www.mdpi.com/article/10.3390/life16050820/s1, Figure S1: General experimental protocol; Figure S2: Uncropped images of the membrane used for p22*^phox^* detection; Table S1: Change in the optical density at 405 nm with or without CD142 antibody.
