# Supplementary material for: Ice-Cold Temperature Enhances NADPH Oxidase-Dependent Release of Tissue Factor-Bearing Extracellular Vesicles from Human Monocytic Cells
Source: Life (Basel). 2026 May 15;16(5):820. doi: 10.3390/life16050820 (PMC13208649; doi:10.3390/life16050820)
Supplement: Supplementary file 1 [file life-16-00820-s001.zip › life-4260522-supplementary.pdf]

Supplemental materials: Involvement of NADPH oxidase in the shedding of tissue factor-bearing vesicles from human monocytic cells exposed to ice-cold temperature

Akira Nishioka [1,2], Toshiharu Azma [1,3], Tsutomu Mieda [3], and Yasushi Mio [4]

1. Department of Anesthesiology and Pain Medicine, Kohnodai Hospital, National Center for the Global Health and Medicine
2. Anesthesiology and Perioperative Medicine, The Jikei University Graduate School of Medicine
3. Department of Anesthesiology, Saitama Medical University Hospital
4. Department of Anesthesiology, The Jikei University School of Medicine

Contents

1. Fig. S1. General experimental protocol
2. Fig. S2. Uncropped images of western blot membranes
3. Table S1. Change in the optical density at 405 nm with or without CD142 antibody

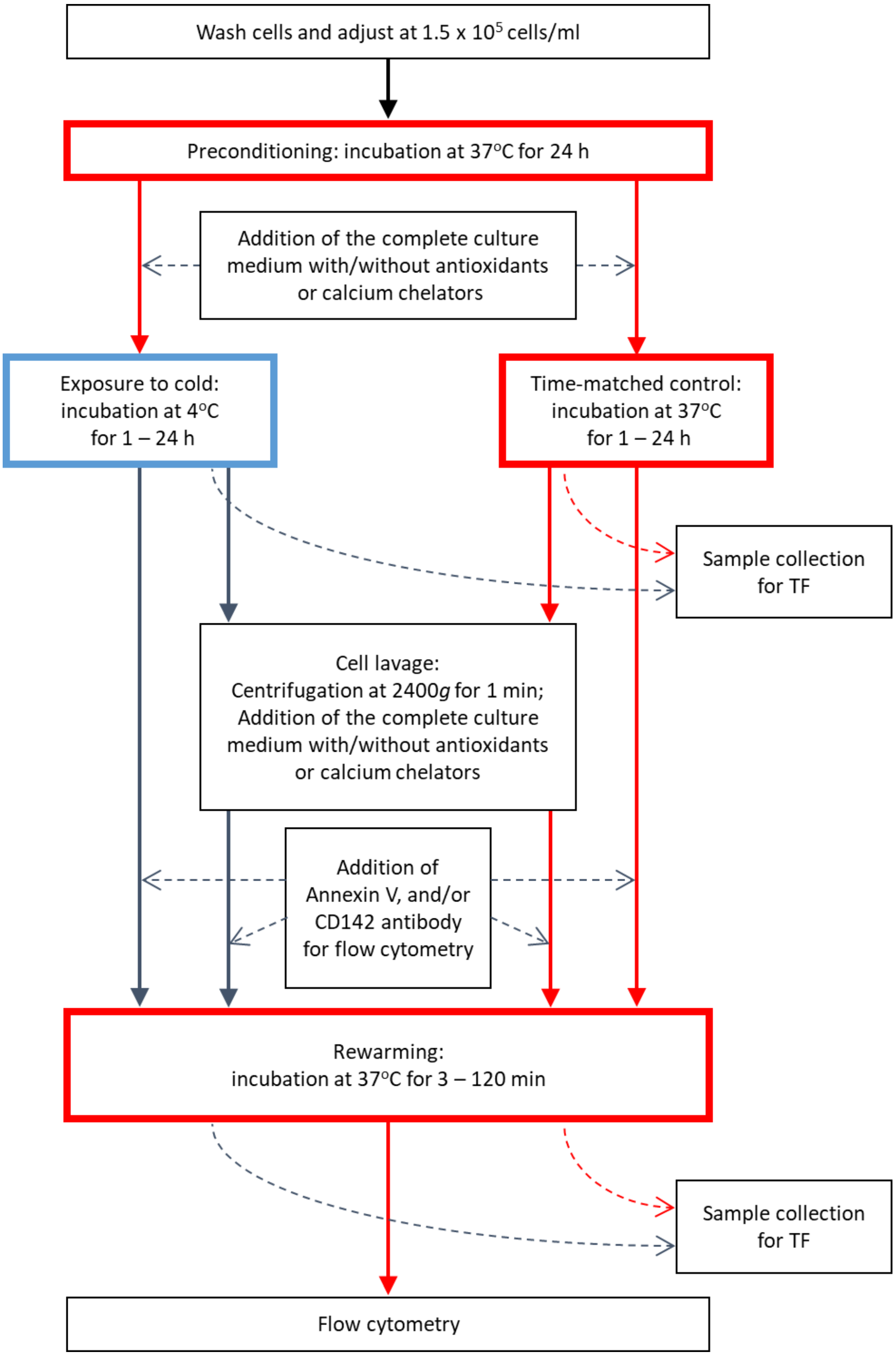

Fig. S1. General experimental protocol

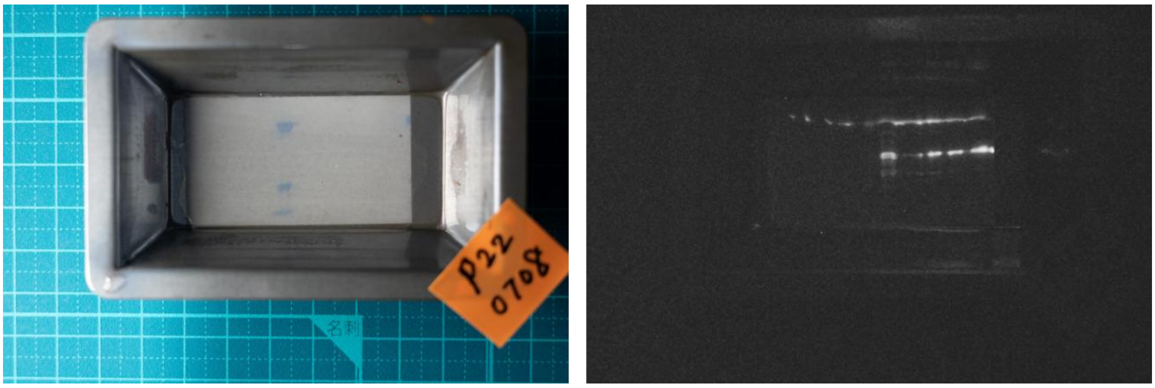

A B

Fig. S2. (A) Uncropped image of the membrane used for p22^{phox} detection under white light. The imaged membrane corresponds to the region used for analysis in Fig. 5. Molecular weight markers (10, 15, and 25 kDa) are indicated. (B) Uncropped chemiluminescence image of the same membrane as shown in (A), used for detection of p22phox. The imaged membrane corresponds to the blot presented in Fig. 5.

Table S1. Change in the optical density at 405 nm with or without CD142 antibody

Sample	(n)	(arbitrary unit/min)
37°C	(4)	2.51 ± 0.05 *
37°C + CD142 antibody	(4)	2.12 ± 0.02 *#
Culture medium	(4)	-0.08 ± 0.02

Changes in the optical density at 405 nm of samples from the supernatant of THP-1 cells incubated at 37°C for 24 h were measured using a multiplate reader in the absence (37°C) or the presence of CD142 antibody (37°C + CD142 antibody). "n" represents the number of samples examined. Data are expressed as mean ± SD. *Significantly different from the "culture medium" (i.e., culture medium was added in the well of multiplate reader instead of the supernatant) ($P < 0.01$). #Significantly different from "37°C" ($P < 0.01$).
